# Supplementary material for: Effects of Paclitaxel on Plasma Membrane Microviscosity and Lipid Composition in Cancer Cells
Source: Int J Mol Sci. 2023 Jul 29;24(15):12186. doi: 10.3390/ijms241512186 (PMC10419023; doi:10.3390/ijms241512186)
Supplement: Supplementary file 1 [file ijms-24-12186-s001.zip › ijms-2507868-supplementary.pdf]

Supplementary Material: Effects of paclitaxel on plasma membrane microviscosity and lipid composition in cancer cells

Liubov Shimolina, Alexander Gulin, Alexandra Khlynova, Nadezhda Ignatova, Irina Druzhkova, Margarita Gubina, Elena Zagaynova, Marina K. Kuimova and Marina Shirmanova

We additionally measured the microviscosity 48 hours after removal of the drug from the culture medium to test the persistence of the action of paclitaxel on the cell membrane. Membrane microviscosity was found to remain low, 271 ± 23 cP, in the absence of the drug (Fig. S1).

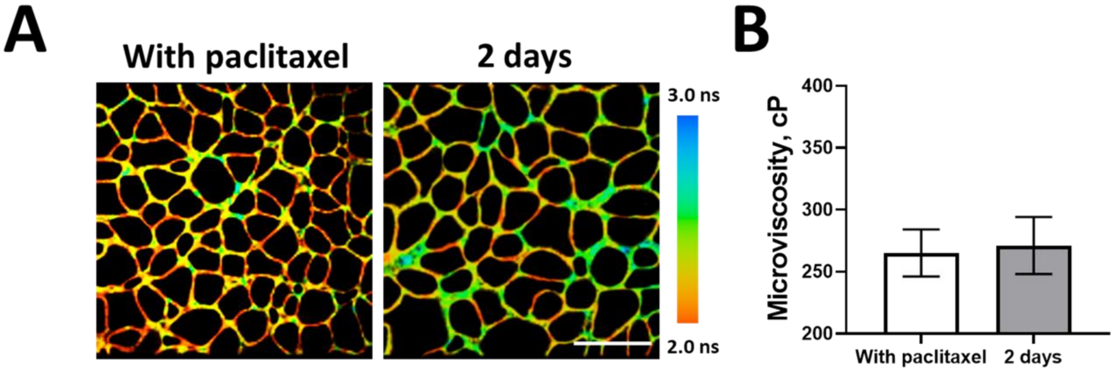

Figure S1. Microviscosity of HeLa cells with and without paclitaxel in the medium. (**A**) Representative FLIM images of cells stained with BODIPY 2 with paclitaxel in the medium and in 2 days after removal of the drug from the medium. (**B**) Quantification of plasma membrane microviscosity. Mean±SD, n = 80 cells. Bar, 40 µm.

The results of the PCA showed only minor differences between control and 24 h for both the positive and the negative ions (Fig. S2).

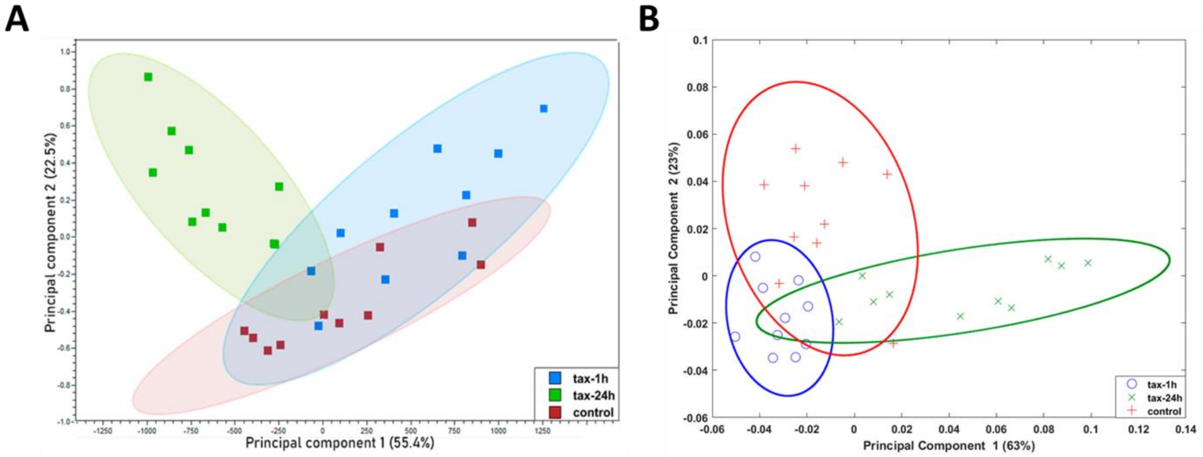

Figure S2. Principal component analysis of ToF-SIMS data demonstrating the differences in membrane lipid composition during paclitaxel incubation. The data from untreated and paclitaxel-treated (1 or 24 h incubation) cells were analyzed. (**A**) Scores plots on PC1 and PC2 resulting from PCA in positive ion mode. (**B**) Scores plots on PC1 and PC2 resulting from PCA in negative ion mode. A 95% confidence limit for each region was defined by an ellipse with the same color to the corresponding region clusters. Each data point represents an individual field of view. Different samples are color coded.

The results of measurements of viscosity and lipid composition in cancer cells after paclitaxel treatment are summarized in Table S1.

Table S1. Summary of the changes in membrane viscosity and lipid profile of cancer cells upon treatment with paclitaxel

	Viscosity	PC	SM	Chol	MUFA	PUFA
Paclitaxel 1 h	=	↑ *	=	=	↑ *	↑ *
Paclitaxel 24h	↓ *	=	↑ *	↑ *	↑ *	↑ *

PC—phosphatidylcholine, SM—sphingomyelin, Chol—cholesterol, MUFA—monounsaturated fatty acids, PUFA—polyunsaturated fatty acids; = equal to control, ↓—lower than control, ↑—higher than control, *—statistically significant difference. Concordant viscosity and lipid data are marked with blue color.

The results of measurements of viscosity and lipid composition in cancer cells after treatment with different drugs are summarized in Table S2.

Table S2. Summary on the effects of drugs on membrane viscosity and the lipid profile of cancer cells upon treatment

	Viscosity	PC	SM	Chol	MUFA	PUFA
Paclitaxel 1 h	=	↑ *	=	=	↑ *	↑ *
Paclitaxel 24h	↓ *	=	↑ *	↑ *	↑ *	↑ *
Cisplatin 1h	↓	↓	=	↑	↓	↓
Cisplatin 24 h	↑ *	↑ *	↑ *	↑ *	↓	↓
Oxaliplatin 1 h	↓ *	↓ *	↓ *	↑ *	↓	↓
Oxaliplatin 24 h	↑ *	↓ *	↓ *	↑ *	↓ *	↓
5-fluorouracil 1h	↑ *	↓ *	↓ *	↓ *	↓	↓ *
5-fluorouracil 24 h	=	↑ *	↑ *	↓ *	↓	↓ *

PC—phosphatidylcholine, SM—sphingomyelin, Chol—cholesterol, MUFA—monounsaturated fatty acids, PUFA—polyunsaturated fatty acids; = equal to control, ↓—lower than control, ↑—higher than control, *—statistically significant difference. Concordant viscosity and lipid data are marked with blue color.
